# Brain Functional Network Generation Using Distribution-Regularized Adversarial Graph Autoencoder with Transformer for Dementia Diagnosis

**DOI:** 10.32604/cmes.2023.028732

**Published:** 2023-08-03

**Authors:** Qiankun Zuo, Junhua Hu, Yudong Zhang, Junren Pan, Changhong Jing, Xuhang Chen, Xiaobo Meng, Jin Hong

**Affiliations:** 1 School of Information Engineering, Hubei University of Economics, Wuhan, 430205, China; 2 State Key Laboratory of Simulation and Regulation of Water Cycle in River Basin, China Institute of Water Resources and Hydropower Research, Beijing, 100038, China; 3 School of Computing and Mathematic Sciences, University of Leicester, Leicester, LE1 7RH, UK; 4 Shenzhen Institutes of Advanced Technology, Chinese Academy of Sciences, Shenzhen, 518055, China; 5 Faculty of Science and Technology, University of Macau, Macau, 999078, China; 6 School of Geophysics, Chengdu University of Technology, Chengdu, 610059, China; 7 Laboratory of Artificial Intelligence and 3D Technologies for Cardiovascular Diseases, Guangdong Provincial Key Laboratory of South China Structural Heart Disease, Guangdong Provincial People’s Hospital (Guangdong Academy of Medical Sciences), Southern Medical University, Guangzhou, 519041, China; 8 Medical Research Institute, Guangdong Provincial People’s Hospital (Guangdong Academy of Medical Sciences), Southern Medical University, Guangzhou, 519041, China

**Keywords:** Adversarial graph encoder, label distribution, generative transformer, functional brain connectivity, graph convolutional network, dementia

## Abstract

The topological connectivity information derived from the brain functional network can bring new insights for diagnosing and analyzing dementia disorders. The brain functional network is suitable to bridge the correlation between abnormal connectivities and dementia disorders. However, it is challenging to access considerable amounts of brain functional network data, which hinders the widespread application of data-driven models in dementia diagnosis. In this study, a novel distribution-regularized adversarial graph auto-Encoder (DAGAE) with transformer is proposed to generate new fake brain functional networks to augment the brain functional network dataset, improving the dementia diagnosis accuracy of data-driven models. Specifically, the label distribution is estimated to regularize the latent space learned by the graph encoder, which can make the learning process stable and the learned representation robust. Also, the transformer generator is devised to map the node representations into node-to-node connections by exploring the long-term dependence of highly-correlated distant brain regions. The typical topological properties and discriminative features can be preserved entirely. Furthermore, the generated brain functional networks improve the prediction performance using different classifiers, which can be applied to analyze other cognitive diseases. Attempts on the Alzheimer’s Disease Neuroimaging Initiative (ADNI) dataset demonstrate that the proposed model can generate good brain functional networks. The classification results show adding generated data can achieve the best accuracy value of 85.33%, sensitivity value of 84.00%, specificity value of 86.67%. The proposed model also achieves superior performance compared with other related augmented models. Overall, the proposed model effectively improves cognitive disease diagnosis by generating diverse brain functional networks.

## 1 Introduction

The brain is an information-processing system with many complicated and precise computations when dealing with various daily activities [1]. Daily physiological activities are always associated with the interaction between multiple neurons, neuronal clusters, or multiple brain regions. This interaction is called brain functional network (BFN), which describes the relationship between temporal blood-oxygen-level-dependent (BOLD) signals from distant brain areas [2]. Dementia (i.e., Alzheimer’s Disease, AD) is a typical kind of neuropathic disorder where patients usually show abnormal functional connections between brain regions [3]. It can result in a series of cognitive symptoms: memory impairment, poor language expression, and changes in vision [4,5]. Functional magnetic resonance imaging (fMRI) can easily capture these abnormal features using the non-intrusive scanning technology [6]. The BFN can bring a new way for the diagnosis and analysis of neurodegenerative disorders [7]. Therefore, analysis of BFNs is important to mine complex brain connectivity features and help detect dementia-related biomarkers. It is further important to understand the pathogenic mechanism and the drug discovery for neurodegenerative disorders [8].

The BFN is usually constructed through a software toolbox by splitting the human brain into predefined Region-of-Interests (ROIs) [9]. The element in the BFN matrix indicates the functional correlation between two ROIs. Many approaches based on machine learning were utilized to diagnose neurological disease in an end-to-end scheme [10–16]. For example, Meier et al. [17] applied the support vector machine (SVM) classifier to distinguish older adults from younger adults using functional connectivity data. To boost the Mild Cognitive Impairment (MCI) prediction performance, Yu et al. [18] designed a sparse graph representation learning method with a weighting scheme to generate sparse BFNs. Bi et al. [19] combined convolutional learning and recurrent learning to extract regional connectivity and adjacent positional features, which proves its learning ability in AD diagnosis. More advanced techniques are proposed to explore the complex connectivity-based features [20–22]. Ji et al. [23] devised novel convolutional kernels to capture hierarchical topological characteristics by element-wise weighting brain networks and achieved more accurate classification performance. The work in [24] applied the graph convolutional network (GCN) method to improve the classification accuracy by jointly using the functional time series and connectivity-based matrices. Nevertheless, the limited medical data makes data-driven models challenging to achieve good prediction results.

The straightforward way to improve classification performance is to synthesize more similar medical data and feed it to data-driven models [25,26]. Numerous data-augmented methods have been developed to solve the small data problem in the field of brain imaging analysis. For example, Hong et al. [27] augmented the routine brain magnetic resonance (MR) imaging with scaling, rotation, translation, and gamma correction and achieved accurate results predicting children’s brain age through a deep learning model. Hu et al. [28] synthesized the positron emission tomography (PET) from MR using generative models, which can handle the problem of incomplete modalities and is promising for multimodal fusion. However, the above methods cannot be applied to BFN augmentation, because it only considers the local features between adjacent pixels and ignores the topological information between distant pixels. Many efforts have been tried to generate new graph data in the graph domain. Meszlényi et al. [29] created some simulated connectivity-based datasets by applying noise weights (NW) to improve the classification performance. The study of [30] solved the problem of small-size data by employing the synthetic minority over-sampling technique (SMOTE) algorithm and achieved a good classification accuracy of non-tumorous facial pigmentation disorders. However, these methods do not directly generate new graph data but interpolate existing brain networks to augment the data, which brings some noise and may have some side effects on the model’s classification performance.

The generative adversarial networks (GANs) [31] is a two-player game that can produce quite good results by mutual game learning [32]. It has gained broad applications in analyzing medical imaging because of its strong ability in distribution fitting [33]. These applications cover the image-related fields, including cross-modal synthesis [34], point cloud generation [35], image super-resolution [36], disease classification [37–39], regression task [40,41], and organ segmentation [42]. Besides, the prior distribution can guide the model’s optimization and thus stabilize the representation learning in the GAN’s training [43]. Reference [44] introduced a Gaussian distribution to constrain the graph embedding in adversarial learning and achieved good performance in graph analytics. The GAN-based model has been applied in the BFN augmentation. For example, Tan et al. [45] utilized the Gaussian noise to synthesize BFNs by applying a semi-positive definite manifold constraint. Also, the transformer network [46] can greatly improve image classification performance by combining adversarial strategy, which can model a strong relationship between distant ROIs.

Motivated by these observations, in this study, a novel distribution-regularized adversarial graph autoencoder (DAGAE) model is proposed to generate BFNs for dementia diagnosis. The main works of this paper are as follows: (1) The label distribution is estimated to regularize the latent space learned by the graph encoder, which can make the learning process stable and deduce a robust representation. (2) The transformer-based network in the generator is introduced to map the node representations into node-to-node connections by exploring the global connectivity information between distant ROIs. It preserves the main topological properties and more discriminative features. (3) The generated BFNs enhance disease prediction using different classifiers, which can be applied to analyze other related brain diseases.

## 2 Materials and Methods

### 2.1 Data Preparation

The purpose of the Alzheimer’s Disease Neuroimaging Initiative (ADNI) project^1^ is to detect the early stage of Alzheimer’s disease from clinical, imaging, gene, biomarker, and other aspects. In this study, we mainly focus on the Late Mild Cognitive Impairment (LMCI) stage scanned with functional Magnetic Resonance Imaging (fMRI). To eliminate the influence of category imbalance on model classification performance, we selected the same number of NC subjects as LMCI for the experiment. About 150 subjects with fMRI were selected to test our model’s effectiveness, including 75 Normal Controls (NC) and 75 LMCI. The fMRI data were scanned with the filed strength of 3.0 Tesla. The turning angle is 80 degrees, and the time of repetition (TR) is in the range of 0.607~3.0 s. The scanning time for each subject is about 10 min.

The commonly used GRETNA [47] software is adopted to preprocess the fMRI to construct graph data. The detailed procedures include format conversion, first ten volumes removal, slice timing, head motion realign, normalizing, spatially smooth, detrend, and temporally filtering (usually 0.01∼0.08 Hz). At last, the automated anatomical labeling (AAL) atlas [48] with ninety non-overlapping ROIs is warped to the fMRI volumes for obtaining functional features *F*. The *F* with the size 90 × 187 is transformed into a BFN *A_ori_* with the dimension size 90 × 90 by the Pearson coefficient algorithm.

### 2.2 Distribution-Regularized Adversarial Graph Autoencoder

**Figure 1:**
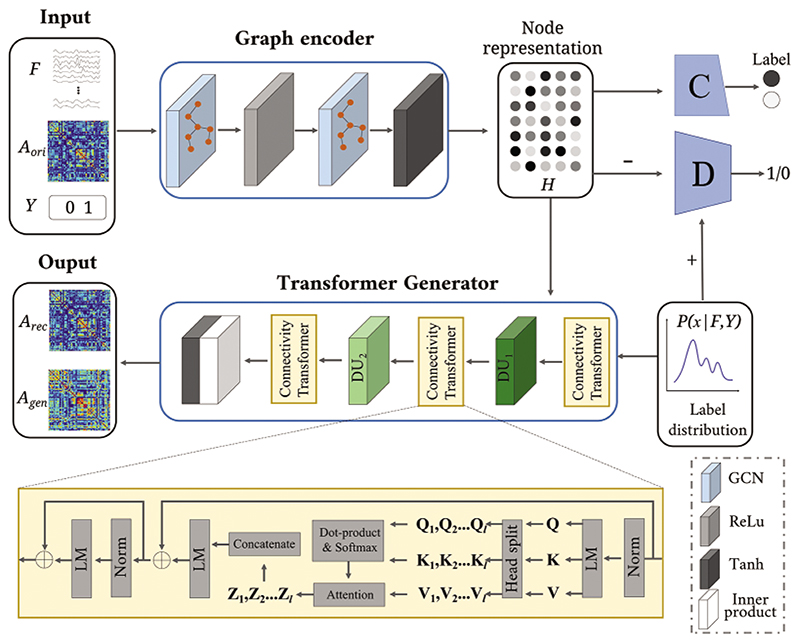
The architecture of the proposed DAGAE model. It accepts brain functional feature *F* and BFN *A_ori_* with a specific label and outputs reconstructed or generated BFN (i.e., *A_rec_*, *A_gen_*). The model has four parts: Graph encoder, classifier, discriminator, and transformer generator

The BFN is generated by the designed DAGAE model, which is depicted in Fig. 1. It accepts the graph data (including brain functional feature *F* and BFN *A_ori_*) and corresponding label (i.e., *Y* = {0, 1}), outputs the reconstructed brain network *A_rec_*, and the generated brain network *A_gen_*. The proposed DAGAE contains three parts: the label distribution estimation (LDE), the adversarial graph encoder (AGE), and the transformer generator (TG). The LDE module is devised to compute the probability distribution of latent node features, which can robustly constrain the graph encoder for node representation learning. The TG maps latent node space to graph space, which decodes the node representations to BFNs. Four objective functions are utilized to optimize the model, including adversarial loss, reconstruction loss, node-representation consistent loss, and cross-entropy loss.

#### 2.2.1 Label Distribution Estimation

To improve the performance of node representation in latent space, the label probability distribution estimated by Kernel Density Estimation (KDE) is introduced in the node representation learning. Instead of the traditional normal distribution, it can reflect the accurate distribution of node features and ensure robust representations in the model training. In the feature space, the node feature *F* ∈ ℝ^*N*×187^ is first passed through a dimension reduction operation to get *H* = {*h*_1_, *h*_2_, …, *h_N_*} ∈ ℝ^*N*×*p*^, and then sent to the KDE for distribution estimation. *N* means the number of brain regions, *p* is the dimension of *h_i_*. The estimated label distribution *P*(*x*|*F*, *Y*) is defined as:

(1) $$P(x|F,Y)=\frac{1}{Nn_{Y}b}\sum_{i=1}^{Nn_{Y}}K(\frac{x-h_{i}}{b})$$

where, *n_Y_* is the subject number with specfic disease (i.e., *Y* = 0 means the NC, *Y* = 1 means the LMCI) subjects. *K*(·) is a predefined kernel function (i.e., Gaussian), and *b* means the kernel’s bandwidth.

#### 2.2.2 Adversarial Graph Encoder

The graph encoder accepts both *F* and *A_ori_* and outputs the latent node representation *H*. To make the node representation learning stable, a prior distribution is introduced to guide the learning process. The graph encoder **E** consists of two GCN layers, where each layer is followed by an activation function. The output dimension of GCN layers is 64 and 32, respectively. The first and second activations are the *ReLU* and *tanh* functions, respectively. The graph encoder can be expressed as:

(2) $$H=\text{E}(A_{ori},F)=GCN_{2}(GCN_{1}(A_{ori},F))$$

The node representation *H* is treated as a fake sample to send to the discriminator **D**.The loss function of the graph encoder in adversarial training is:

(3) $$L_{enc\;}=E_{F∼P_{fMRl},A_{ori\;}∼P_{fMRI}}[log(1-D(E(A_{ori\;},F)))]$$

**Figure 2:**
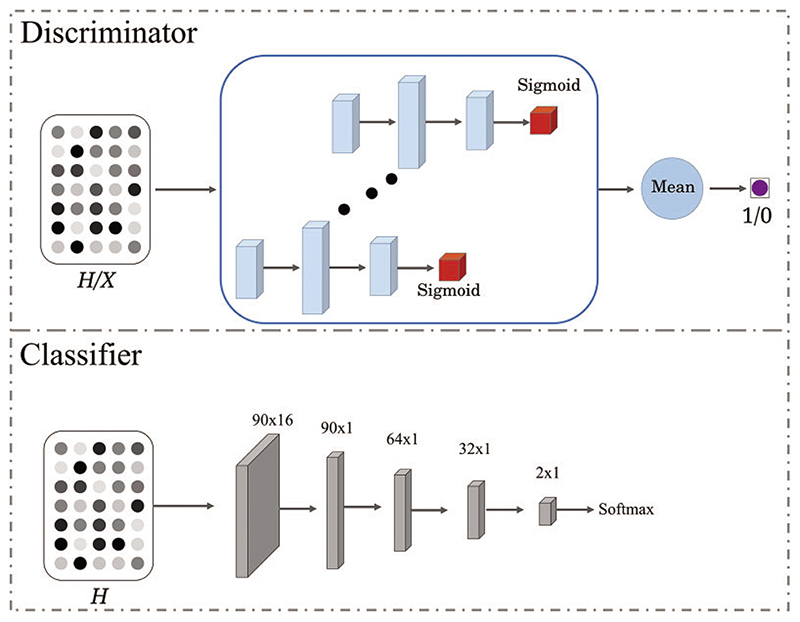
Illustration of the discriminator and classifier structure. The input of the discriminator is a matrix computed from either latent node representation *H* or label distribution *P_x_*, and the output is true (1) or false (0)

The graph encoder aims to enforce the latent node representation *H* to be consistent with the estimated label distribution *P_x_*. The estimated label distribution *P_x_* is utilized to guide the graph encoder to learn a robust representation. The discriminator plays as a referee to supervise the graph encoder to learn a distribution-consistent representation. Specifically, we sample a matrix *X* ∈ ℝ^*N*×*p*^ from the distribution *P_x_*. The sampled matrix *X* is the real sample for adversarial learning, while the fake sample is the output *H* of the graph encoder. As shown in Fig. 2, the discriminator comprises *N* sub-networks, where each discriminates the true or false of only one ROI representation. Each subnetwork is built on a three-layer perceptron with hidden neurons 32, 64, and 1. Each subnetwork consists of a *sigmoid* activation function to keep the output in the range of 0∼1. The output of the discriminator is the mean value of all the subnetwork outputs. The discriminator loss is:

(4) $$L_{dis\;}=E_{F∼P_{fMRI},A_{ori\;}∼P_{fMRl}}[-log(1-D(E(A_{ori\;},F)))]+E_{X∼P_{x}}[-log(D(X)))]$$

In addition, to make the node representation discriminative, the cross-entropy loss is introduced to further regularize the learned node representation. The binary classifier **C** is shown in the lower part of Fig. 2. The node representation *H* passes five Multi-Layer Perceptron (MLP) and outputs a vector with two elements, followed by a softmax to predict the most likely disease category. The classifier loss can be computed as follows:

(5) $$L_{cla\;}=E_{F∼P_{fMRl},A_{ori\;}∼P_{fMRl}}[y\cdot log(C(E(A_{ori\;},F)))]$$

here, the *y* is a one-hot vector (i.e., [0,1] represents the LMCI, and [1,0] represents NC).

#### 2.2.3 Trasnformer Generator

The transformer generator **G** process maps each node in latent space *H* to a reconstructed graph *A_rec_*. Also, the **G** can generate similar brain networks *A_gen_* by inputting matrix *X* sampled from the label distribution *P_x_*. The generator module comprises three connectivity transformer (CT) layers, two dimensions upscaling (DU) layers, and connectivity prediction operation. The connectivity transformer layer contains norm, linear mapping (LM), head split, attention, dot-product, and concatenate. 4, 8, and 11 heads are designed in the three successive CT layers. Note that each CT’s input and output dimension is the same. The output dimension of the two DU layers is 64 and 187, respectively. Each DU has only one layer. After latent representation *H* passes through the CT and DU layers, the inner product and *tanh* activation function are utilized to predict connectivity with the range −1∼1. The reconstructed BFN *A_rec_* and generated BFN *A_gen_* are given by:

(6) $$A_{rec\;}=G(H)=tanh(H^{\prime }\cdot H^{\prime T})$$

(7) $$H^{\prime }=CT(DU_{2}(CT(DU_{1}(CT(H)))))$$

(8) $$A_{gen\;}=G(X)=tanh(X^{\prime }\cdot X^{\prime T})$$

(9) $$X^{\prime }=CT(DU_{2}(CT(DU_{1}(CT(X)))))$$

The reconstruction loss is adopted to preserve the original graph structure while making the autoencoder training stable. We choose the L1 norm to measure the distance between the original *A_ori_* and reconstructed *A_rec_*. It is defined as:

(10) $$L_{rec}=E_{A_{ori}\sim P_{fMRI},F\sim P_{fMRI}}(||\text{G}(\text{E}(A_{ori},F))-A_{ori}|{|}_{1})$$

Moreover, to make the generator learning more stable, we put the reconstructed brain network *A_rec_* to the graph encoder and obtain consistent node representation $\hat{H}.$ The node-representation consistent loss is calculated by minimizing the distance generated between *H* and $\hat{H}:$

(11) $$L_{nrc}=E_{H∼P_{H}}(∥\hat{H}-H{∥}_{1})$$

(12) $$\hat{H}=\text{E}(\text{G}(H))$$

### 2.3 Classification Training and Evaluation Metrics

In summary, the optimization strategy of the proposed DAGAE updates the weights of the graph encoder, discriminator, classifier, and transformer generator. The hybrid loss is defined by:

(13) $$L_{all}=L_{enc\;}+L_{dis}+L_{cla}+L_{rec}+L_{nrc}$$

**Figure 3:**
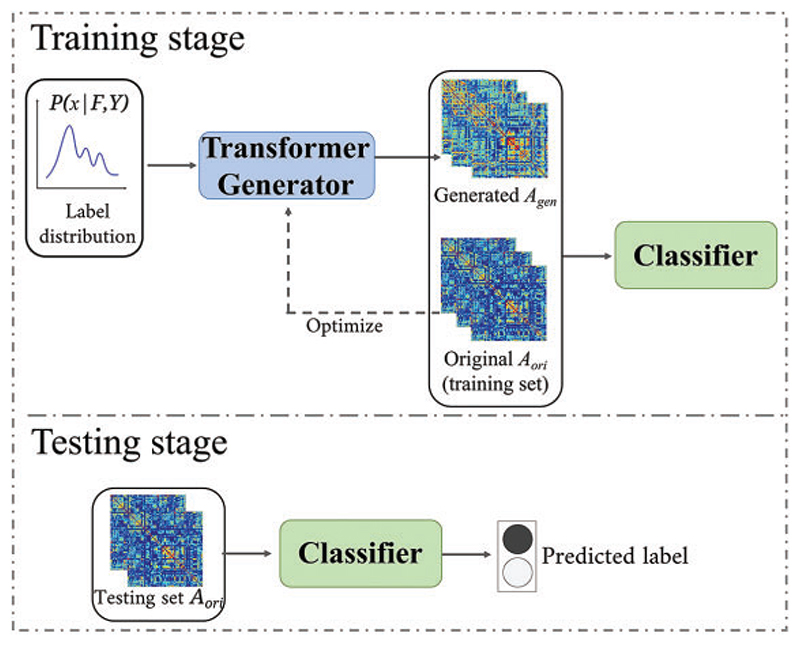
The entire workflow of this work. In the training stage, the transformer generator is first extracted from the trained DAGAE and then maps the distribution-sampled representation into generated BFNs (*A_gen_*). At last, the combination of the generated and original BFNs is utilized for training the classifier. In the testing stage, only the original BFNs in the testing set are used to predict the disease label

As illustrated in Fig. 3, we send the training set to the DAGAE model for training and augment the BFN with the transformer generator. The detailed training of the DAGAE model is shown in Algorithm 1. Inspired by the method [49,50], the latent feature learning can be stable when the optimization converges. For each label (i.e., NC and LMCI), we sample matrics from the distribution *P_x_* and generate *k* times the number of original BFNs. It should be noted that the generated BFNs are not seen in the test set.

In the classification stage, we build a sample graph classifier modified from [24], including two GCN layers with 32 and 16 hidden neurons, one graph pooling, and one MLP layer with two neurons. The original training set is first used to train the graph classifier. Then the generated BFNs are mixed in the training set to finetune the classifier for enhancing classification performance. At last, the trained classifier predicts disease labels of the testing set for performance evaluation. There are four commonly used metrics for the prediction assessment: Accuracy (ACC), Specificity (SPE), Sensitivity (SEN), and the Area Under the receiver operating characteristic Curve (AUC). They are defined as:

(14) $$ACC=\frac{TL+TN}{TL+TN+FL+FN}$$

(15) $$SEN=\frac{TL}{TL+FN}$$

(16) $$SPE=\frac{TN}{TN+FL}$$

where, *TN* means that NC is correctly predicted, *TL* means that LMCI is correctly predicted. *FN* means that NC is incorrectly predicted, *FL* means that LMCI is incorrectly predicted.

Algorithm 1 **Optimizing the DAGAE model**

**Input:**   *F*: brain functional feature;

            *A_ori_*: original BFN;

            *P_x_*(*F*, *Y*): label distribution;

            *O*: the number of iterations;

            *T*: the number of steps for updating discriminator

**Output:**    *A_gen_* ∈ ℝ^*N*×*N*^ : the generated BFN

1:  **for** *i* = 1, 2, …, *O* **do**

2:      **for** *j* = 1, 2, …, *T* **do**

3:          Compute the latent representation matrix *H* using Eq. (2)

4:          Sample a true matrix *X* = {*x*_1_, *x*_2_, …, *x_N_*} from the label distribution *P_x_*{*F*, *Y*}

5:          Compute the loss function $L_{dis}$

6:          Update the discriminator **D** by propagating the gradient $\nabla L_{dis\;}^{j}[log(D(X))+log(1-D(H))]$

7:      **end for**

8:      Compute the combined loss function $ℒ={ℒ}_{enc}+{ℒ}_{cla}+{ℒ}_{rec}+{ℒ}_{nrc}$

9:      Update the encoder, classifier, and generator by back-propagating the gradient$-\nabla L^{i}$

10:     Compute the generated BFN $A_{gen}^{i}$ using Eq. (8)

11:     Replace the generated *A_gen_* with $A_{gen}^{i}$

12:  **end for**

## 3 Experiment and Results

### 3.1 Experimental Setup

We adopt the 5-fold cross-validation strategy in the experiment to conduct the training and testing. The preprocessed data is evenly separated into five folds, meaning each fold contains 15 NCs and 15 LMCIs.We first selected one-fold data and sent the rest of the four folds data (60 NCs and 60 LMCIs) into the DAGAE model for training. Next, the trained generator is used to generate *k* (default value 1.0) times the training data. Then the generated and original training data are jointly to train the classifier. Finally, the classifier predicts the disease label of the selected one-fold data for performance evaluation. In this experiment, three other augmented methods (i.e., EW[29], SMOTE [30] and ARAE [44]) and three classical classifiers (i.e., SVM [51], DNN [52], and GCN [53]) are introduced to test the effectiveness of the proposed model.

The DAGAE is trained on Ubuntu18.04 using the TensorFlow framework for BFN synthesis. The graphical device is one GPU with NVIDIA Quadro P4000 8.0 GB. We set the model’s parameters with the values as follows: *N* = 90, *p* = 32. In the DAGAE training, we first update the weighting parameters of the graph encoder, classifier, and discriminator and then optimize the generator parameters. The learning rate for the encoder and the discriminator is set at 0.001 and 0.0001, respectively. The learning rate of the classifier and the generator are the same as the discriminator. The Adam algorithm is selected for training with batch size 16. The learning process terminates when the discriminator cannot identify the input from the node representation or the prior label distribution and the change of total loss is stable. After the DAGAE has been trained, the generator is used to augment BFNs for training the classifier. The learning rate of the classifier is set as 0.0001. We took about 1000 epochs for training to ensure the classifier’s convergence. The ACC value is defined as the classification performance evaluation, which is used to optimize the classifiers.

### 3.2 Prediction Performance

**Figure 4:**
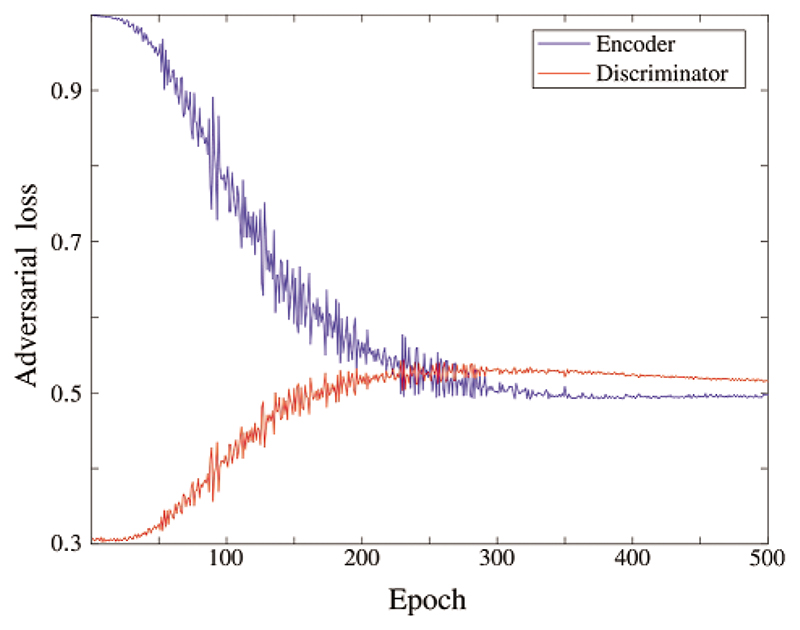
The loss curve of the adversarial training. It is utilized to constrain the latent node representations in the label distribution

**Figure 5:**
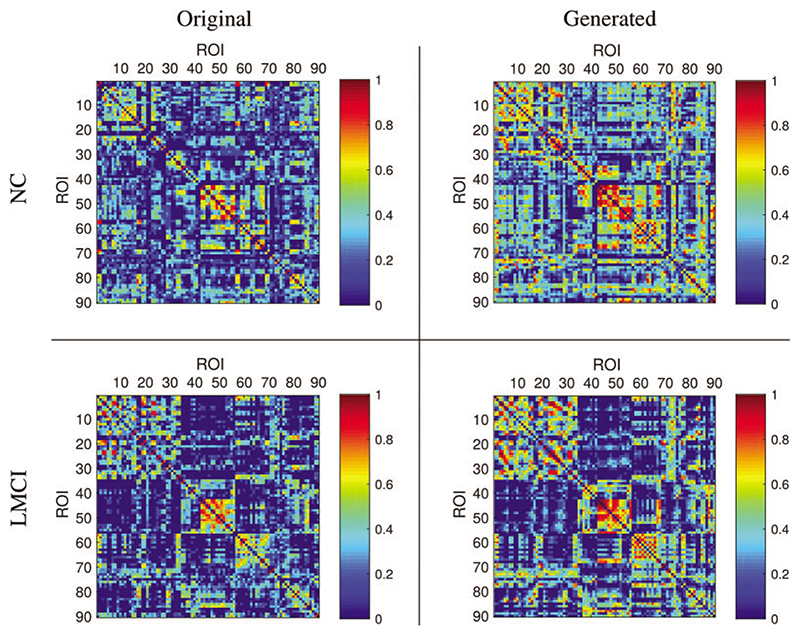
Visualization of the original and generated BFNs. The left column shows the original BFNs at NC and LMCI stages, and the right column shows the generated BFNs at NC and LMCI stages

In the experiment, it is essential to constrain the latent node representation to follow the label distribution. This constraint can diminish the model overfitting and stabilize the representation learning. Fig. 4 shows the adversarial loss over epochs in the training process. In the beginning, the encoder loss falls, and the discriminator rises. After 250 epochs, both keep around 0.5 steadily, which means the adversarial training converges. After the training, the transformer generator generates new BFNs by accepting node representation matrices sampled from the label distribution *P_x_*. As shown in Fig. 5, examples of the original and generated BFNs are compared qualitatively. It can be seen that the generated BFN can preserve the main patterns of the original BFN.

**Figure 6:**
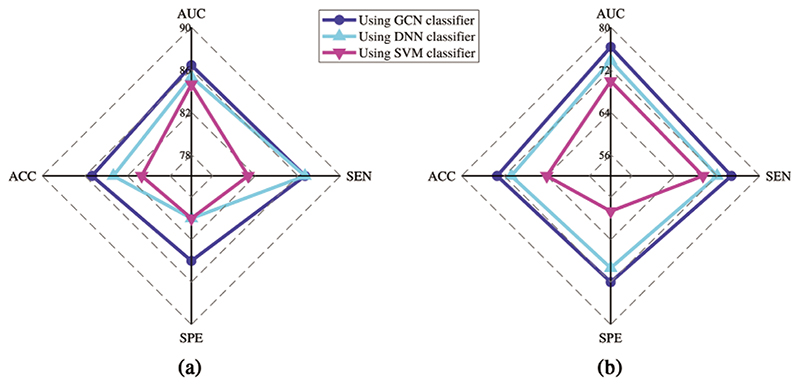
The prediction performance comparison using three classifiers by inputting BFNs from (a) our model, (b) original

**Figure 7:**
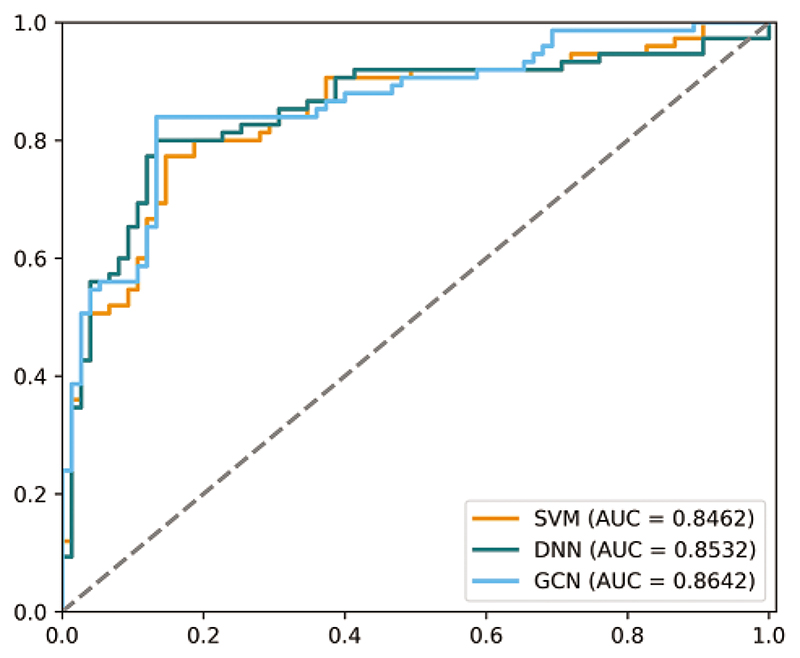
The comparison of ROC curves using three classifiers. The gray dotted line represents the random classifier

We analyze the classification performance with different classifiers to investigate the proposed model’s effectiveness. As shown in Table 1, the generated BFNs can gain better classification performance over original BFNs. Among the three augmented methods, our model achieved superior results in three classifiers with more than 10 percent of ACC value compared to results using original BFNs. Also, our model increases the ACC value by 1.3%, 2.0%, and 3.3% compared with the competing ARAE method for GCN, DNN, and SVM classifiers, respectively. This evidence proves that the proposed model can generate more effective BFNs for classification improvement. To detail the effectiveness of the GCN-based classifier, Figs. 6a and 6b show that the prediction results using a GCN-based classifier achieves the best performance than other traditional classifiers. Note that both original and generated BFNs using different methods are sent to the same classifier for classification performance evaluation. Fig. 7 also shows better performance of the GCN-based classifier. Our model shows superior prediction performance in terms of ACC, SEN, SPE, and AUC by achieving 85.33%, 84.0%, 86.67%, and 86.42%. It probably indicates that the GCN-based classifier can benefit the topological properties buried in the BFNs and enhance the classification of BFNs.

### 3.3 Evaluation of the Generated BFNs

**Figure 8:**
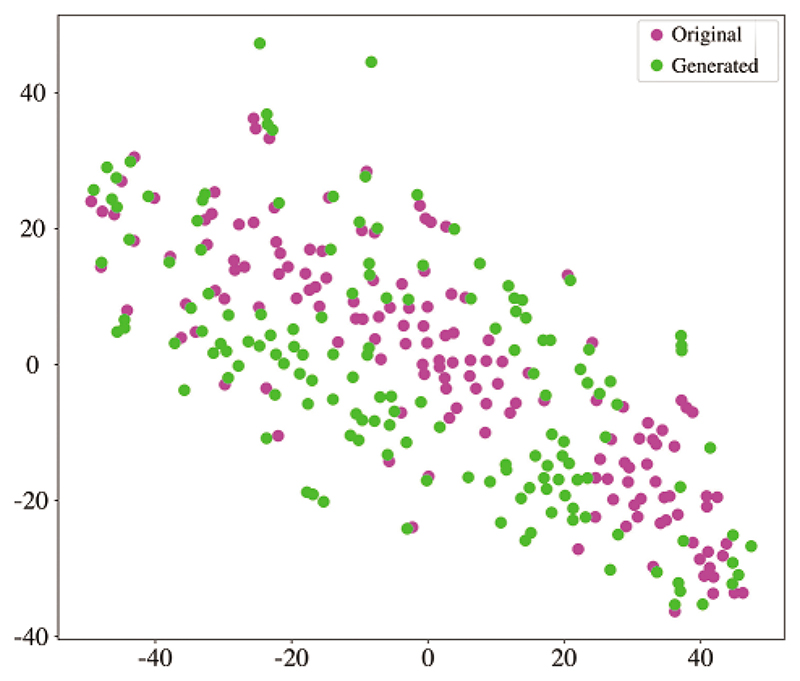
The comparison of embedded t-SNE representation between the original and generated BFNs

**Figure 9:**
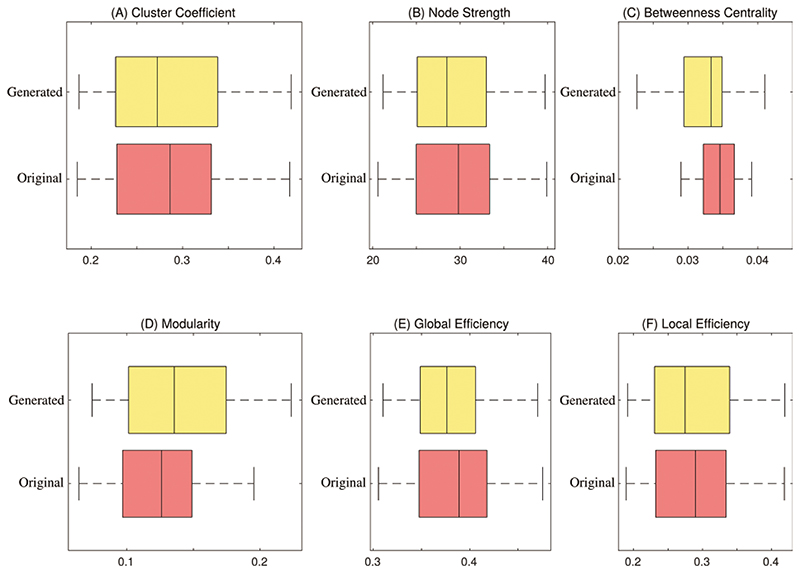
Statistical analysis of the original and generated BFN. (A) Cluster coefficient, (B) Node strength, (C) Betweenness centrality, (D) Modularity, (E) Global efficiency, and (F) Local efficiency

In this section, we evaluate the coherence between original and generated BFNs. We generated the same size as the original data in each one-fold training. We employed the t-distributed Stochastic Neighbour Embedding (t-SNE) tool [54] to analyze the graphical characteristics. Fig. 8 shows the projection of the embedding representation of original and generated BFNs from high-dimensional space to two-dimensional space. The generated data is consistent with the original data distribution, which ensures the similarity between the generated and original FBNs. In addition, six common metrics are utilized to quantitatively measure the effectiveness of the generated data. These six metrics can provide a relatively reliable measure of generated BFN’s quality, including clustering coefficient, node strength, betweenness centrality, modularity, local efficiency, and global efficiency. As shown in Fig. 9, the boxplot distribution of each metric is compared between the generated and original BFNs. The generated data can mostly cover the range of graph metrics from the original data. Therefore, the generated BFNs by our model contains non-Euclidean characteristics and preserve the overall nature of brain connectivity, which is suitable to augment the BFNs for dementia diagnosis.

## 4 Discussion

**Figure 10:**
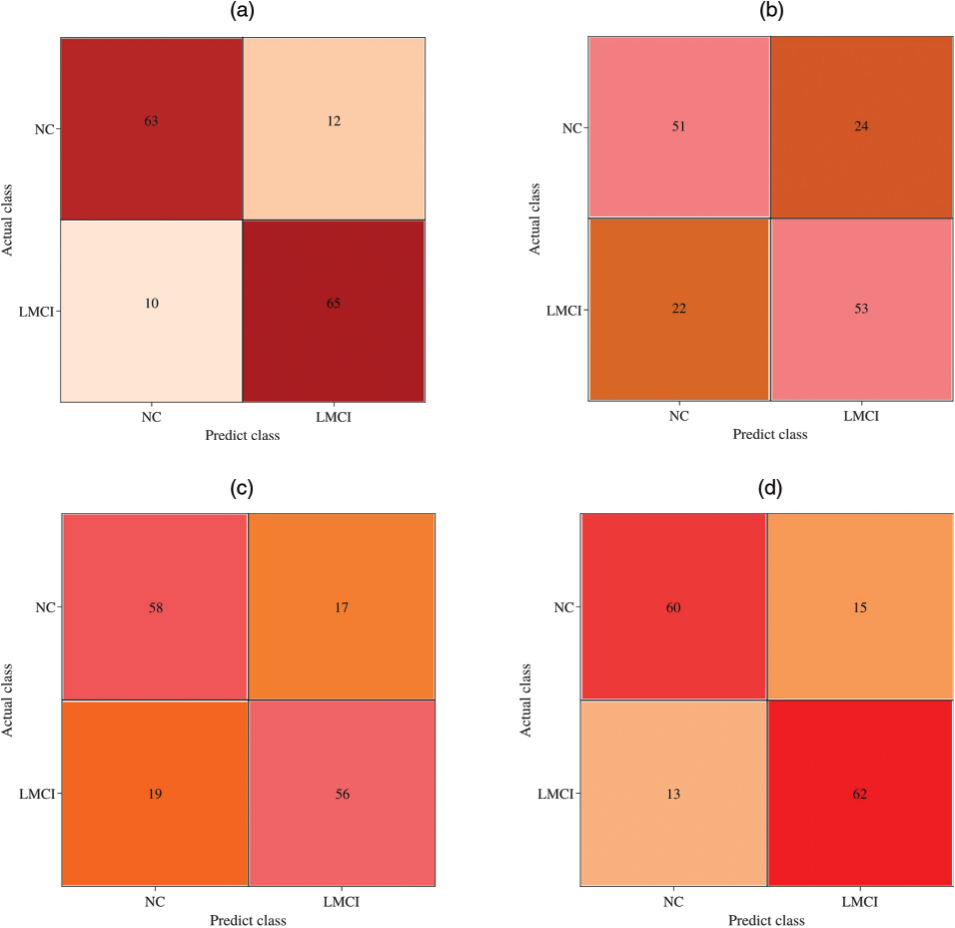
Influence of different modules on the classification performance. (a) The proposed DAGAE, (b) DAGAE without encoder, (c) DAGAE without discriminator, and (d) DAGAE without the classifier

**Figure 11:**
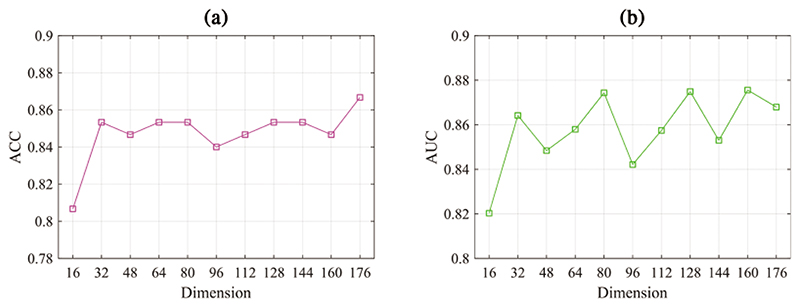
Impact of the dimension *p* of the learned node representation *H* on the ACC and AUC

The proposed DAGAE model can generate new BFNs for improving classification performance. Each module in the model contributes to the generation quality of BFNs. To analyze the influence of different modules, we remove the encoder, discriminator, and classifier from the DAGAE model and evaluate the final classification performance. Fig. 10 demonstrates that the encoder significantly impacts the whole model. It drops by 16% in terms of ACC by removing the encoder module. The discriminator ensures the latent node representation in a prior distribution, which also influences the quality of the generated BFNs. This suggests the usefulness of prior label distribution can regularize the latent representation with a stable learning strategy and enhance the BFN classification. Furthermore, we study the dimension *p* of the latent node representation *H* in the prediction performance. As shown in Fig. 11, the value of ACC and AUC shows relatively stable fluctuation when *p* exceeds 32. Considering the computation efficiency, we select *p* = 32 in the experiment. The transformer generator is essential for the generated BFN quality. We study two variations of the designed transformer generator to test its effectiveness. (1) Remove the DU layer in the generator (No-DU), which means the input and output dimension is the same as the dimension of latent representation *H*; (2) remove the connectivity transformer (No-CT), which simplifies the generator into two layer perceptrons. The results are illustrated in Table 2; it can be seen that the combination of DU and CT achieves the best prediction performance. The classification results demonstrate the effectiveness of the transformer generator in the model. This can be explained by that the transformer-based network in the generator preserves the main topological properties and captures more discriminative features.

Data-driven models achieve better performance by using large amounts of data. To investigate how much generated data influences the prediction performance, we generate *k* ∈ {1, 2, …, 8} times the original training set, combine the original BFNs and generated BFNs to train the SVM-based and GCN-based classifier. The mean ACC is estimated by predicting five-fold original test sets separately.

**Figure 12:**
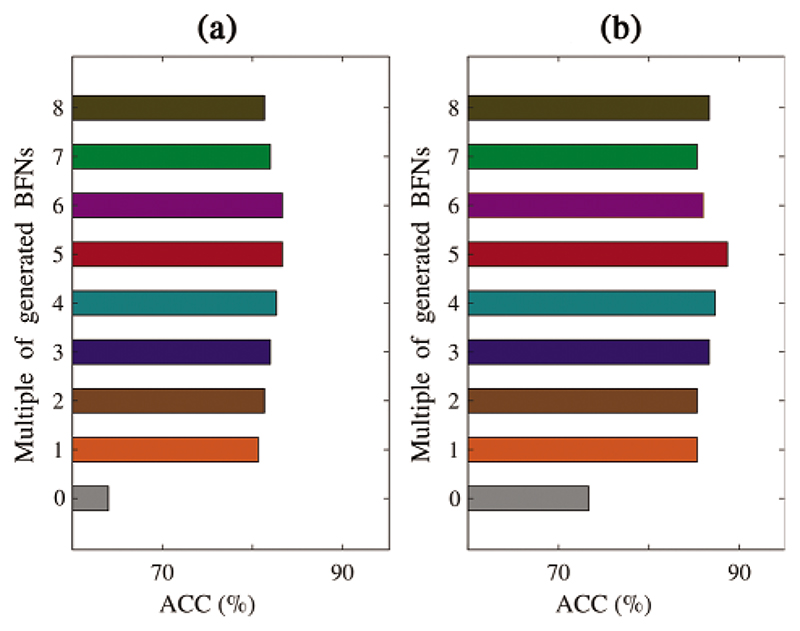
Classification accuracy analysis using different amounts of generated data by our model using (a) SVM and (b) GCN classifier, respectively

The metric *DeltaACC* is defined as *ACC_k_* – *ACC*_0_. Here, *ACC*_0_ refers to the predicted results of the classifier trained using the original train set, and the *ACC_k_* refers to the predicted results of the classifier trained using the original and generated data. Fig. 12 gives the different quantities of generated BFNs that maximizes classification performance in both classifiers. The best quantity of generated data is about five times the original data, with the largest *DeltaACC* value of 19.3% and 13.3% for the SVM and GCN classifier, respectively. The reason why more data degrades classification performance may be that the generated BFNs bring a lot of noise. Besides, compared with the SVM classifier, the GCN-based classifier increases by 5.3% on the best ACC value. This marginal increase may be explained by considering the topological information among the brain regions, which can characterize the disease-related features of the BFNs. Thus, in the BFN augmentation experiment, it is better to choose the GCN-based classifier to evaluate the prediction performance, and different quantities of generated BFNs should be explored to maximize the effect of data augmentation.

Although the proposed DAGAE is promising in augmenting the BFN data for disease prediction, there are still two limitations that have not been considered. (1) The label distribution is estimated using the limited training data, which can not add other prior knowledge. We will introduce disease-related anatomical brain knowledge into the model for performance evaluation. (2) The data in this work are deliberately picked out to maintain category balance. The real condition in the category distribution is always imbalanced. In the following study, we will try to apply the proposed DAGAE in category-imbalanced datasets for other brain disorder diagnosis.

## 5 Conclusions

This study proposes a novel DAGAE model to augment new BFNs for dementia diagnosis. The BFN augmentation is different from traditional image synthesis, where the latter only extracts local patterns and ignores the topological information buried in the brain network. Our model is novel in two aspects. One is that the estimated label distribution can regularize the latent space and make the learning process stable. Another one is that the transformer generator is devised to map the node representations into node-to-node connections by exploring the long-term dependence of highly-correlated distant brain regions, which preserves the main topological properties and more discriminative features. Testing on the Alzheimer’s Disease Neuroimaging Initiative (ADNI) public dataset, the proposed DAGAE can generate similar and high-quality BFNs. The classification results show that adding generated data can achieve higher accuracy values of 85.33%, 83.33%, and 80.67% than the original method using GCN, DNN, and SVM classifiers, respectively. The proposed model also performs better than related augmented models, providing new insight for improving cognitive disease diagnosis accuracy.

## Figures and Tables

**Table 1:** Comparison of classification performance using different generated BFNs

BFNs generated from	Classifier	ACC	SEN	SPE	AUC
Original	SVM	64.00%	58.66%	69.33%	69.85%
EW[29]	SVM	68.00%	70.67%	65.33%	74.63%
SMOTE [30]	SVM	75.33%	78.67%	72.00%	79.70%
ARAE [44]	SVM	77.33%	70.67%	74.00%	79.11%
**Our model**	SVM	**80.67**%	**80.00**%	**81.33**%	**84.62**%
Original	DNN	70.67%	69.33%	72.00%	73.71%
EW[29]	DNN	75.33%	70.67%	79.99%	77.10%
SMOTE [30]	DNN	80.67%	78.67%	82.67%	82.66%
ARAE [44]	DNN	81.33%	77.33%	85.33%	84.44%
**Our model**	DNN	**83.33**%	**80.00**%	**86.67**%	**85.32**%
Original	GCN	73.33%	72.00%	74.67%	76.28%
EW[29]	GCN	80.67%	81.33%	79.99%	82.95%
SMOTE [30]	GCN	82.67%	81.33%	84.00%	83.56%
ARAE [44]	GCN	84.00%	82.67%	85.33%	86.91%
**Our model**	GCN	**85.33**%	**84.00**%	**86.67**%	86.42%

**Table 2:** Effect of different structures in the transformer generator

Method	ACC	SEN	SPE	AUC
Our model	**85.33**%	84.00%	**86.67**%	**86.42**%
No-DU	81.33%	84.00%	78.67%	82.84%
No-CT	78.67%	82.67%	74.67%	82.63%

## Footnotes

1
http://adni.loni.usc.edu/
